# Human T cells expressing BEND3 on their surface represent a novel subpopulation that preferentially produces IL-6 and IL-8

**DOI:** 10.1002/iid3.17

**Published:** 2014-02-19

**Authors:** Hirokazu Shiheido, Koji Kitagori, Chiyomi Sasaki, Shio Kobayashi, Takane Aoyama, Kozue Urata, Takuma Oku, Yoshitaka Hirayama, Hiroyuki Yoshitomi, Masaki Hikida, Hajime Yoshifuji, Tsuneyo Mimori, Takeshi Watanabe, Jun Shimizu

**Affiliations:** 1 Center for Innovation in Immunoregulative Technology and Therapeutics, Graduate School of Medicine, Kyoto UniversityKyoto, Japan; 2 Department of Rheumatology and Clinical Immunology, Graduate School of Medicine, Kyoto UniversityKyoto, Japan; 3 Pharmacology Research Laboratories, Drug Discovery Research, Astellas Pharma Inc.Tsukuba, Japan

**Keywords:** BEND3, IL-6, IL-8

## Abstract

BEN domain-containing protein 3 (BEND3) has no transmembrane region, is localized in the cytoplasm, and is involved in chromatin function and transcription. We here identified a novel subpopulation of human T cells that expressed BEND3 on their cell surface (BEND3^+^ T cells). BEND3^+^ T cells consisted of approximately 3% of T cells in the peripheral blood, were present in both CD4^+^ and CD8^+^ T cells, and were also observed in cord blood. The stimulation of BEND3^+^ T cells through the TCR/CD3 complex led to the production of various kinds of cytokines; however, the levels of IL-6 and IL-8 produced by BEND3^+^ T cells were higher than those by BEND3^−^ T cells. The proportion of BEND3^+^ T cells was also increased in some patients with inflammatory diseases. Taken together, these results indicate that BEND3^+^ T cells are a new subpopulation of T cells in terms of their cytokine profile. Further analyses on BEND3^+^ T cells may be of importance and useful in understanding human T cell immunology.

## Introduction

The immune system consists of many types of cells. Analysing and understanding various immune responses have shown that the identification of novel types of cells, cell subpopulations, and/or cell subsets is very important and useful [1–4]. One of the representative examples is the discovery of regulatory T cells (Treg cells) as a T cell subset [5]. Many advantages may be obtained in analysing immune systems by classifying CD4^+^ T cells into Treg and non-Treg cells. In addition, it is also important to identify the existence of new subsets/subpopulations that differentiate from naïve cells [6–8]. Furthermore, previous studies have demonstrated that a particular cell population could differentiate into a different functional subpopulation [9–11].

The identification of population-specific markers to distinguish a cell population from others is of importance in studies investigating cell population. Proteins on the cell surface are often used as a marker, while intracellular proteins are also useful [12]. In this study, we demonstrated that BEND3 could be used to identify a new subpopulation in human T cells. BEND3, a protein containing four BEN domains with no transmembrane region, is thought to be involved in chromatin function and transcription. Previous studies reported that BEND3 is a heterochromatin-associated protein [13–15].

We here showed that T cells in the peripheral blood expressed BEND3 intracellularly, and also that a small proportion of T cells expressed BEND3 on their surface (BEND3^+^ T cells). BEND3^+^ T cells exclusively produced significantly higher levels of IL-6 and IL-8 upon stimulation than those produced by BEND3^−^ T cells. The proportion of BEND3^+^ T cells was stable in healthy donors, but was variable in some patients with inflammatory diseases.

## Results

### A small proportion of T cells in human PBMCs were stained with #16-15 Ab

**Figure 1:**
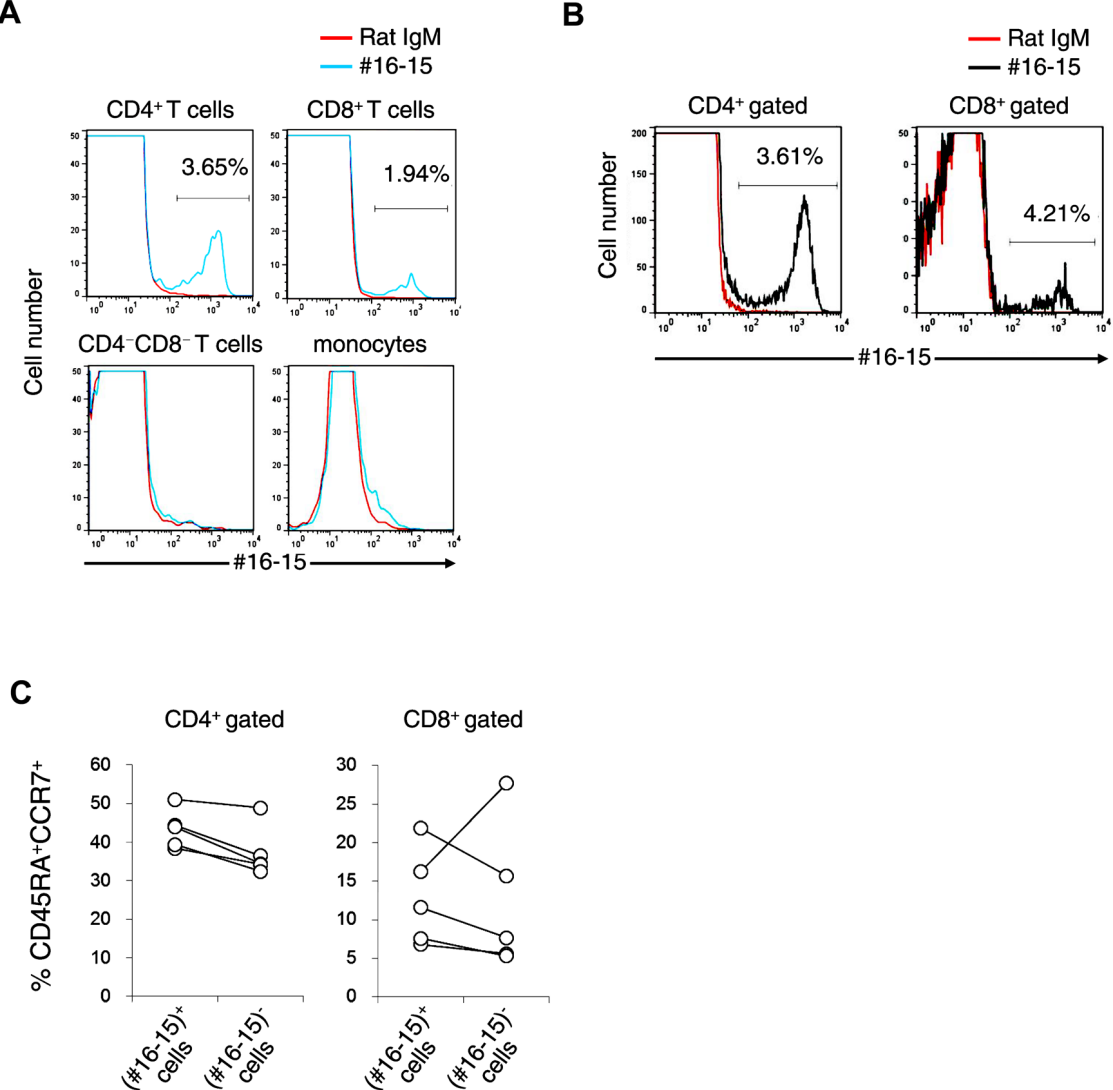
Staining of human T cells with #16–15. (A) PBMCs from healthy donors were stained with anti-CD4, anti-CD8 Abs, 7AAD, and #16-15 or rat IgM. Cells stained with #16-15 or rat IgM as a control Ab in CD4^+^ T cells, CD8^+^ T cells, CD4^−^CD8^−^ cells, or monocytes (gated in FSC-SSC dot-blot) were shown as a histogram. (B) Human cord blood cells were stained and analyzed as in (A). Results are representative of more than 10 (A) and three (B) independent experiments. (C) The percentage of CD45RA^+^CCR7^+^ T cells in (#16-15)^+^ or (#16-15)^−^ T cells in PBMCs from healthy donors was plotted. Each symbol represents different individuals. Data from the same donor were linked with lines (*n* = 5). *P* = 0.136 or 0.935, the proportion of CD45RA^+^CCR7^+^ T cells in CD4^+^ or CD8^+^ T cells, respectively, in (#16-15)^+^ versus (#16-15)^−^ T cells.

Rats were immunized with CD4^+^ T cells prepared from fresh healthy human peripheral blood mononuclear cells (PBMCs), and spleen cells were fused with myeloma cells. Supernatants (SNs) from the resulting hybridomas were screened for their ability to stain the human T cell line, Jurkat. One of the established monoclonal antibodies (Ab), #16-15 (rat IgM), could stain Jurkat cells (data not shown), but exhibited unique staining for PBMCs. (#16-15)^+^ cells consisted of a small proportion of T cells in human PBMCs (in CD4^+^ T cells, 3.52 ± 0.74%, *n* = 30; in CD8^+^ T cells, 2.96 ± 1.61%, *n* = 30; Fig. 1A). (#16-15)^+^ T cells were also observed in cord blood, and the proportion of (#16-15)^+^ cells in CD4^+^ or CD8^+^ T cells was similar to that in the peripheral blood of healthy adults (Fig. 1B). We also examined the proportion of naïve or memory T cells in (#16-15)^+^ or (#16-15)^−^ cells. No obvious skewing was observed in the proportion of naïve (Fig. 1C) or memory (Fig. S1) T cells between (#16-15)^+^ and (#16-15)^−^ cells.

### #16-15 Ab recognized BEND3 on the cell surface

**Figure 2:**
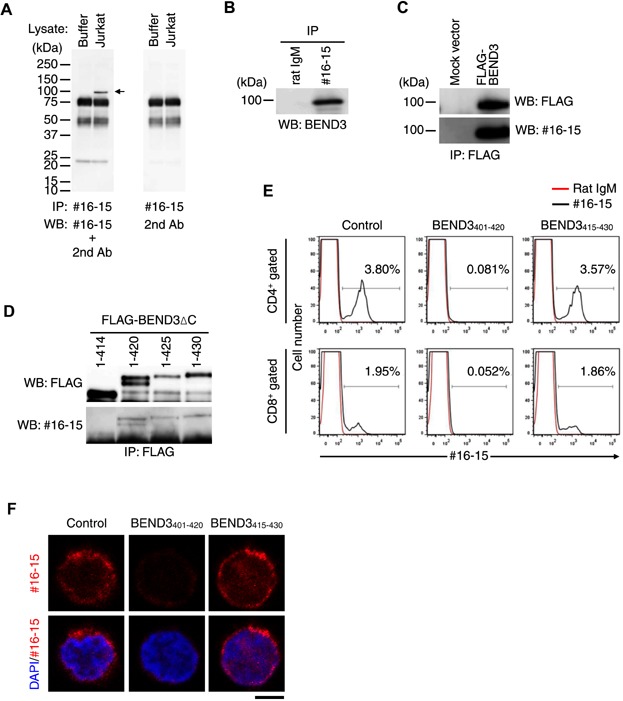
#16-15 recognized BEND3. (A) The lysis buffer or lysate prepared from Jurkat cells was immunoprecipitated (IP) with #16-15-conjugated beads. The resulting immunoprecipitated proteins were separated in SDS–PAGE, and blotted (WB) with #16-15. Arrow: target molecule recognized by #16-15. (B) The lysate from Jurkat was immunoprecipitated with #16-15, and blotted with the anti-BEND3 Ab. (C) Jurkat cells were transfected with a mock or FLAG-BEND3 expression vector. Cell lysates were subjected to immunoprecipitation (IP) using anti-FLAG Ab. Immunoprecipitates were separated by SDS–PAGE under reduced conditions. The blot was probed with an anti-FLAG Ab or #16-15. (D) Jurkat cells were transfected with the FLAG-tagged and C-terminus-truncated BEND3 expression vector (FLAG-BEND3ΔC). The lysate was immunoprecipitated with the anti-FLAG Ab, and blotted with the anti-FLAG Ab or #16-15. (E) PBMCs were stained with #16-15, anti-CD4, and anti-CD8 Abs in the presence or absence of the BEND3 peptide as indicated. Staining with #16-15 in CD4^+^ (top) or CD8^+^ T cells (bottom) was shown as a histogram. (F) Intracellular BEND3 in CD4^+^ T cells prepared from PBMCs was stained with #16-15 in the presence or absence of the BEND3 peptide. Bar = 5 μm. Results are representative of more than three (A–F) independent experiments.

To identify the target molecule recognized by #16-15, lysates prepared from Jurkat cells were immunoprecipitated with #16-15, subjected to SDS–PAGE under reduced conditions, transferred onto a membrane, and blotted with #16-15 (Fig. 2A). The resulting specific band with a molecular weight of 100 kDa was subjected to mass spectrometry analysis. A subsequent database search revealed BEND3 (828 a.a. in humans) as the target molecule. This result was confirmed in the following experiments; A Jurkat-derived and #16-15-immunoprecipitated protein was blotted with the anti-BEND3 polyclonal Ab (Fig. 2B); FLAG-tagged BEND3 precipitated with the anti-FLAG Ab was also blotted with the anti-FLAG Ab and #16-15 (Fig. 2C). These results demonstrated that #16-15 recognized BEND3.

We then attempted to identify the determinant on BEND3 recognized by #16-15 using Jurkat cells transfected with a vector expressing BEND3, which was FLAG-tagged at the N-terminus and truncated at the C-terminus (FLAG-BEND3ΔC). FLAG-BEND3_1–420_ and longer ones were immunoprecipitated with the anti-FLAG Ab and blotted with #16-15 (Fig. 2D). In the case of FLAG-BEND3_1–420_, FLAG-BEND3 was always observed as a doublet. The reason why FLAG-BEND3_1–420_ did this remains unclear. In contrast, although FLAG-BEND3_1–414_ was immunoprecipitated with the anti-FLAG Ab, it was not recognized by #16-15. Collectively, these approaches revealed that the determinant was contained in BEND3_1–420_, but was not or was partially contained in BEND3_1–414_. Two peptides (BEND3_401–420_ and BEND3_415–430_) derived from BEND3 were prepared based on these results.

Staining human T cells with #16-15 in the presence of the BEND3_401–420_ peptide, but not BEND3_415–430_, was inhibited (Fig. 2E), which demonstrated that the determinant existed within BEND3_401–420_. BEND3 was initially reported to be localized intracellularly [13]. #16-15 was able to stain intracellular BEND3 in human T cells, and this staining was also inhibited with the BEND3_401–420_ peptide (Fig. 2F), which indicated that #16-15 could stain BEND3 existing both intracellularly and on the cell surface. Collectively, these results demonstrated that T cells in the peripheral blood expressed BEND3 intracellularly, and that a small proportion of T cells also expressed BEND3 on their surface.

We investigated whether a mouse T cell subpopulation existed that was similar to human BEND3^+^ T cells. The sequence of BEND3_401–420_ recognized by #16-15 was completely conserved between human and mouse BEND3. This prompted us to investigate whether #16-15 recognized mouse BEND3. #16-15 could stain intracellular BEND3 in mouse T cells (Fig. S2A). This staining was inhibited with the BEND3_401–420_ peptide, but not with BEND3_415–430_, which demonstrated that #16-15 could cross-react with mouse BEND3. However, staining normal mouse spleen cells with #16-15 did not reveal the presence of T cells expressing BEND3 on the cell surface (Fig. S2B). BEND3^+^ T cells were not detected in any case even though staining was performed using normal mouse cells from the lymph nodes, Peyer's patch, thymus, and peripheral blood, or T cells from inflammation-induced mice (Fig. S2C–E). Thus, T cells expressing BEND3 on the cell surface appear to be unique to human T cells.

### BEND3^+^ T cells preferentially produced IL-6 and IL-8

**Figure 3:**
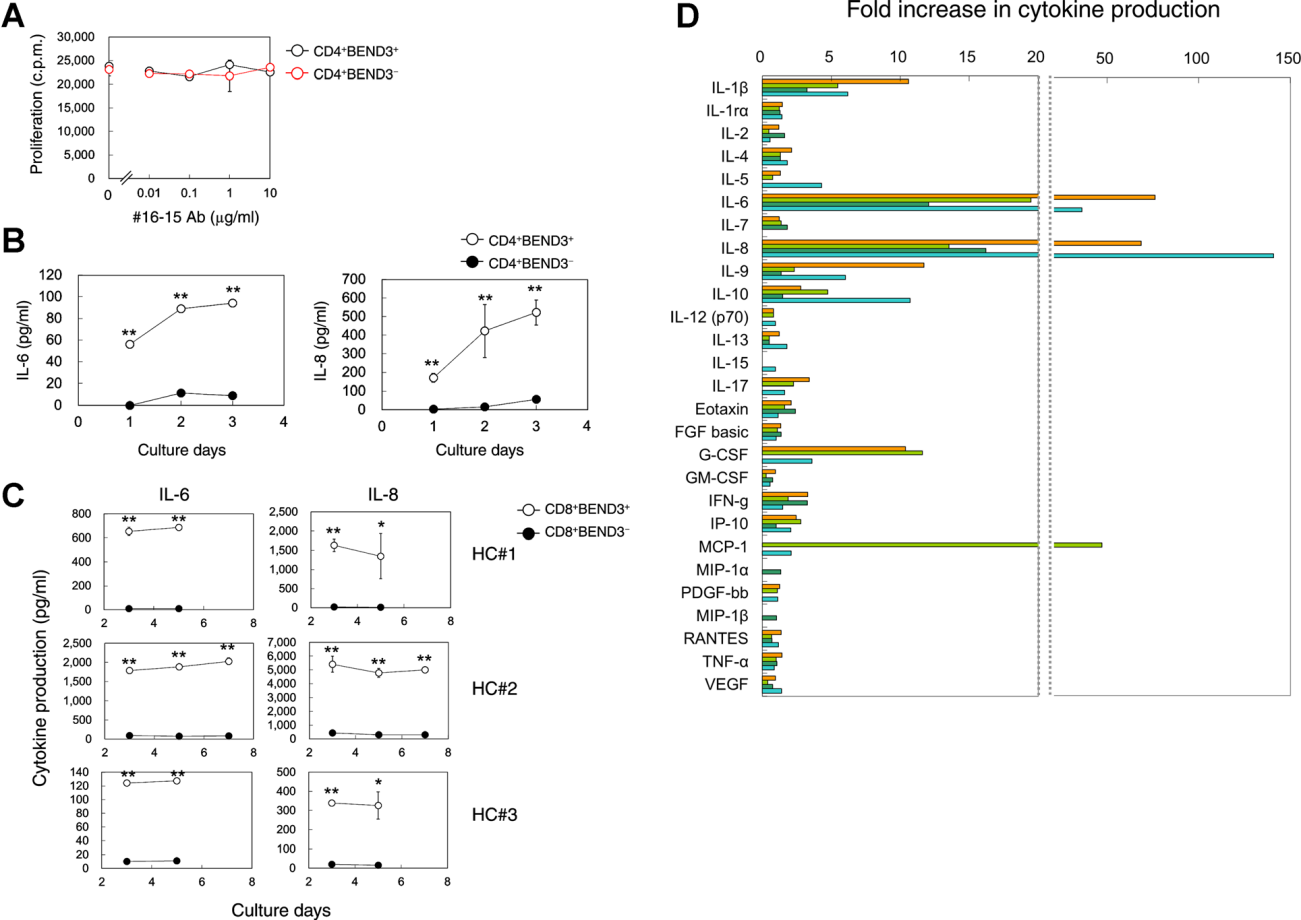
Markedly higher production of IL-6 and IL-8 by BEND3^+^ T cells. (A) CD4^+^BEND3^+^ or BEND3^−^ T cells were sorted from the PBMCs of healthy donors, and stimulated with anti-CD3/CD28 beads in the presence or absence of the titrated amount of #16-15. Cell proliferation was measured 3 days later. Results are expressed as mean counts per minute ±SD of triplicate cultures and are representative of three independent experiments. (B) CD4^+^BEND3^+^ or BEND3^−^ T cells were sorted from PBMCs obtained from healthy donors, and stimulated with anti-CD3/CD28 beads. Culture supernatants were harvested on the indicated days, and the amount of cytokines produced was measured with the Bio-Plex kit. Values are shown as means ± SD; *n* = 3. Results are representative of five independent experiments. **P < 0.01 by the Student's *t*-test. (C) CD8^+^BEND3^+^ or BEND3^−^ T cells were sorted from PBMCs obtained from three healthy control donors (HC#1–#3), and were stimulated with anti-CD3/CD28 beads. Culture SNs were harvested on the indicated days, and the amount of cytokines produced were measured with the Bio-Plex kit. Values are shown as means ± SD; *n* = 3. *P < 0.05, **P < 0.01 by the Student's *t*-test. (D) The amount of cytokines produced by CD8^+^BEND3^+^ T cells was compared with that by CD8^+^BEND3^−^ T cells, and was shown as a fold increase. Fold increase = (the amount of cytokines produced by CD8^+^BEND3^+^ T cells)/(the amount of cytokines produced by CD8^+^BEND3^−^ T cells). The four different colors represent different individuals.

We then investigated whether any functional differences were apparent between BEND3^+^ and BEND3^−^ T cells. Both cells could be stimulated to the same level with anti-CD3/CD28 beads in proliferation assays, and #16-15 exhibited no inhibitory effect on the proliferation of BEND3^+^ T cells (Fig. 3A). The production of cytokines in these stimulation cultures was then analyzed. The stimulation with anti-CD3/CD28 beads induced the production of various kinds of cytokines in CD4^+^ and CD8^+^ T cells regardless of the expression of BEND3. However, the levels of IL-6 and IL-8 (and IL-1β to some extent, as shown in Fig. 3D) produced by BEND3^+^ T cells were significantly higher than those by BEND3^−^ T cells (Fig. 3B–D). Although the absolute amounts of IL-6 and IL-8 produced were different among healthy individuals (Fig. 3C), the markedly higher production of IL-6 and IL-8 by BEND3^+^ T cells was constantly observed (Fig. 3D).

### The proportion of BEND3^+^ T cells was variable

**Figure 4:**
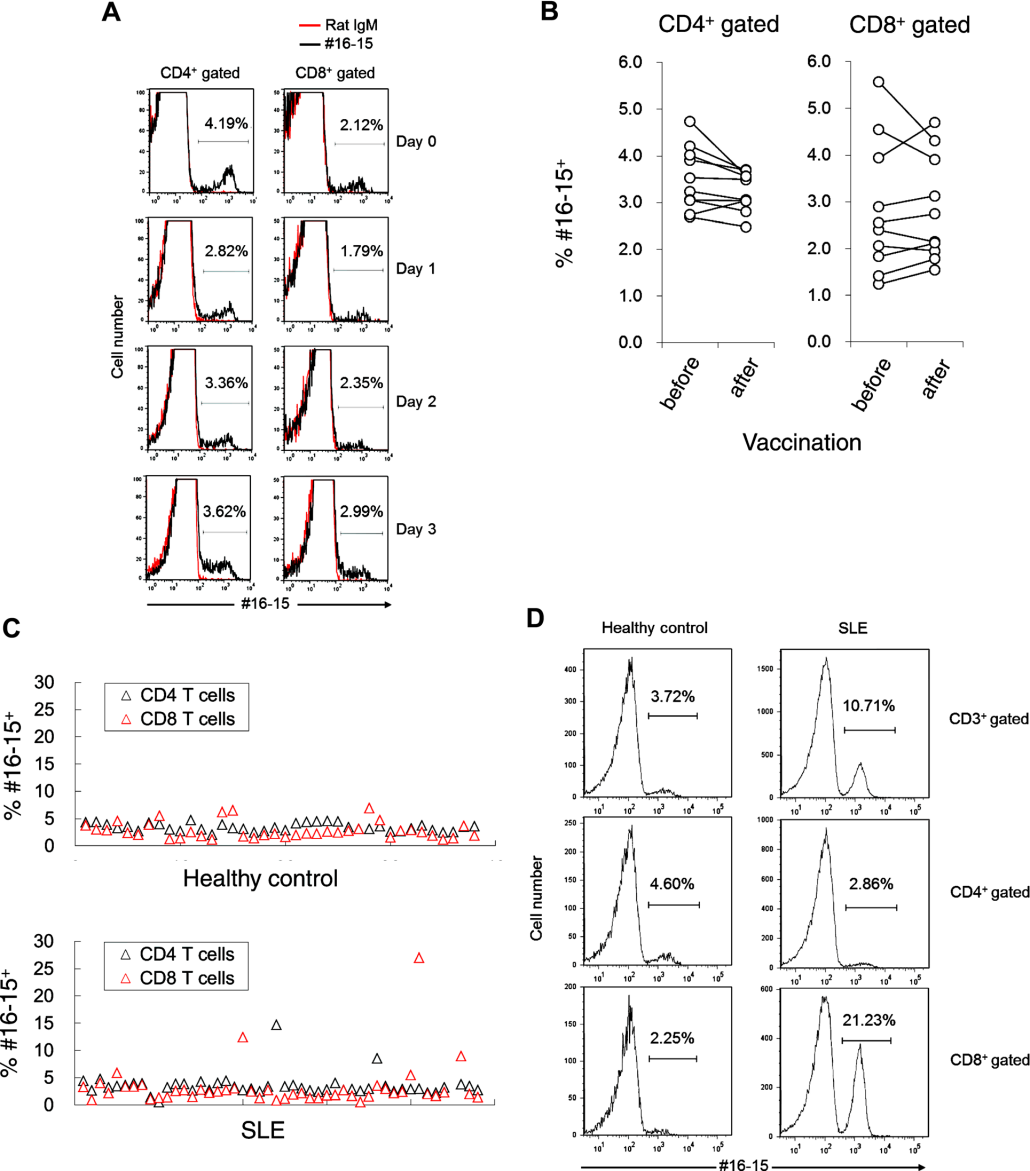
Changes in the proportion of BEND3^+^ T cells. (A) Whole PBMCs from healthy donors were stimulated with anti-CD3/CD28 beads for the indicated time, and were then stained with anti-CD4 Ab, anti-CD8 Ab, #16-15, or rat IgM and 7AAD. BEND3^+^ T cells in CD4^+^ or CD8^+^ T cells were shown as a histogram. Results are representative of three independent experiments. (B) The percentage of BEND3^+^ T cells in CD4^+^ or CD8^+^ T cells from 10 volunteers before and after vaccinations was plotted. Each symbol represents different individuals. Data from the same donor were linked with lines (*n* = 10). (C) The percentage of BEND3^+^ T cells in CD4^+^ or CD8^+^ T cells from healthy donors (*n* = 38) or SLE patients (*n* = 47) was plotted. Each symbol represents different individuals. Data from the same individual were plotted on the same *x*-axis. (D) Staining results from a SLE patient with a high percentage of BEND3^+^ T cells in CD8^+^ T cells were shown as a histogram.

We showed that the proportion of BEND3^+^ cells in T cells appeared to be maintained at the same level in cord blood and PBMCs (Fig. 1). To investigate whether the proportion of BEND3^+^ in T cells was stable, PBMCs were stimulated with anti-CD3/CD28 beads in the presence or absence of various reagents (IFN-γ, IL-4, IL-13, MCP-1, TNF-α, G-CSF, IL-17A, IL-8, LPS, IL-1β, or IL-6), and the proportion of BEND3^+^ T cells was then examined. However, the culture conditions examined had no clear influence on the proportion of BEND3^+^ cells (Fig. 4A).

Whether any changes occurred in the proportion of BEND3^+^ T cells was also examined before and after an influenza vaccination (Fig. 4B). However, clear changes were not observed in this experiment. In contrast, the proportion of BEND3^+^ T cells was high in the peripheral blood of some patients (Fig. 4C and D). For example, a marked increase in BEND3^+^ T cells in CD4^+^ or CD8^+^ T cells was observed in some patients with systemic lupus erythematosus (SLE). However, no correlation was observed between SLE and the increase in BEND3^+^ T cells (*P* = 0.87 or 0.61, the proportion of BEND3^+^ T cells in CD4^+^ or CD8^+^ T cells, respectively, in healthy donors vs. SLE patients). Whether any relationship exists between the proportion of BEND3^+^ T cells and disease needs to be investigated in more detail.

## Discussion

BEND3 contains four BEN domains. The BEN domain has been suggested to mediate DNA–protein or protein–protein interactions [14,15]. In accordance with this proposal, BEND3 was shown to be a heterochromatin-associated protein that repressed transcription [13]. In contrast, in addition to intracellular BEND3, we here reported that BEND3 was also expressed on the surface of T cells (Figs. 1 and 2). The proportion of BEND3^+^ T cells in PBMCs from healthy donors was almost stable (Fig. 4) and similar results were also observed in cord blood (Fig. 1B), which suggested that BEND3^+^ T cells may not be an induced cell population such as Th1, Th2, Th17, and many others [6–8].

BEND3^+^ T cells produced higher levels of IL-6 and IL-8 than BEND3^−^ T cells (Fig. 3). IL-6 is well-known as an important inflammatory and multi-functional cytokine [16]; IL-8 is a chemoattractant for many other inflammatory cells including neutrophils, basophils, and T cells [17–19]. Therefore, the activation of BEND3^+^ T cells, which are able to markedly and abundantly produce both IL-6 and IL-8 upon stimulation through TCR/CD3, may be important for the effective and prompt initiation and promotion of inflammatory responses in the early stage of inflammation. Conversely, abnormal activation and/or dysregulation of BEND3^+^ T cells may be a reason for chronic inflammation.

It is well-known that memory T cells produce higher amount of cytokines than naïve cells upon stimulation [20–23]. Therefore, it may be possible to assume that higher levels of IL-6 and IL-8 production are ascribed to the higher proportion of memory/effector cells in BEND3^+^ T cells than BEND3^−^ T cells. However, we demonstrated that no obvious skewing was observed in the proportion of naïve (Tn) or memory T cells (Tcm, Tem, and Temra) between BEND3^+^ and BEND3^−^ T cells (Figs. 1C and S1). In addition, we observed the markedly higher production of IL-6 and IL-8 only by BEND3^+^ T cells. Regarding other cytokines, we did not observe any significant differences (Fig. 3D). Therefore, it is unlikely that preferential production of IL-6 and IL-8 by BEND3^+^ T cells results from higher content of memory/effector T cells in BEND3^+^ T cells. Whether the higher production of IL-6 and IL-8 is ascribed to a smaller subpopulation within BEND3^+^ T cells needs to be investigated in more detail.

We demonstrated that a significantly higher proportion of BEND3^+^ T cells was observed in some SLE patients (Fig. 4). We compared SLE patients who exhibited a high proportion of BEND3^+^ T cells with those who exhibited a normal proportion in terms of disease activity, organ involvement, and medication. However, we did not observe any particular relationship. Therefore, we need to further investigate whether the proportion of BEND3^+^ T cells can be a marker of a particular disease and/or a particular stage in a particular disease, and what factors cause the increase in BEND3^+^ T cells.

The characteristics of the higher production of IL-6 and IL-8 raise the possibility that BEND3^+^ T cells may be involved in inflammatory responses. However, the physiological roles of BEND3^+^ T cells in vivo remain unclear. The anti-BEND3 Ab (#16-15), which we established in this study, could cross-react with mouse BEND3 (Fig. S2). Nevertheless, we could not observe T cells expressing BEND3 on their cell surface. However, this does not suggest that BEND3^−^ T cells with similar properties (the preferentially higher production of IL-6 and IL-8) to human BEND3^+^ T cells may not be present in mice. The identification of such T cell populations in mice may be useful for understanding the role of this population in vivo. Furthermore, the depletion or expansion of BEND3^+^ T cells may be useful for manipulating immune responses without any side effects on other T cells.

In this study, we showed that BEND3 was localized not only intracellularly, but also on the cell surface. How BEND3, which has no transmembrane domain, localizes on the cell surface and its role on the cell surface remain unknown. High mobility group box 1 (HMGB1), as an example of intracellular proteins, was released, could associate with other proteins on the cell surface, and may provide signals [24]. BEND3 on the cell surface as well as intracellular BEND3 may also bind to various proteins and be involved in many functions. Therefore, it may be possible to regard BEND3 on BEND3^+^ T cells as one of the functional modes of BEND3. Further analyses on BEND3^+^ T cells may be of importance and useful in understanding the role of BEND3 and human T cell immunology.

## Materials and Methods

### Antibodies and reagents

Antibodies against CD4, CD8, CD45RA, CCR7, and rat IgM were all purchased from BD Biosciences; CD3 from eBioscience; FLAG from Sigma; BEND3 from Sigma, Nobus Biologicals, Abcam, or Abgent. Pristine and 7AAD were all purchased from Sigma; thioglycolate from Nissui (Tokyo, Japan); Pertussis toxin from LIST BIOLOGICAL LABORATORIES; the BEND3_401–420_ peptide (LDEASSPGEFAVFLLHRLFP) and BEND3_415–430_ peptide (LHRLFPELFDHRKLGE) from Operon Biotechnology (Japan); cytokines from Miltenyi Biotec, eBioscience, and BD Biosciences.

### Cell preparation

The study protocol was approved by the Review Boards for human studies in Kyoto University. Peripheral blood was obtained from consenting healthy adult donors and SLE patients. Human peripheral blood mononuclear cells (PBMCs) were isolated from the blood using a Ficoll-Paque PLUS (GE Amersham) gradient. CD4^+^ T cells were isolated from PBMCs using magnetic beads conjugated with the anti-CD4 Ab and a magnetic column (Miltenyi Biotec, Gladbach, Germany). To purify T cell subpopulations, PBMCs were stained with anti-CD4, anti-CD8, #16-15 Abs, and 7AAD, and BEND3^+^ or BEND3^−^ subpopulations in CD4^+^ or CD8^+^ T cells were sorted using a flow cytometer (FACSAria, BD Biosciences, San Jose, CA). Cord blood cells were purchased from ALLCELLS, and Jurkat [25] cells were from the American Type Culture Collection (ATCC). BALB/c mice were purchased from Charles River Japan Inc. (Kanagawa, Japan).

### Preparation of monoclonal antibodies

Wistar rats (2 months old, purchased from Japan SLC) were intraperitoneally immunized three times every 2 weeks with CD4^+^ T cells (2 × 10^6^) prepared from fresh healthy human PBMCs, and were intravenously injected with human CD4^+^ T cells (1 × 10^7^) 1 month later. Spleen cells were fused with P3X63Ag8.653 myeloma cells (from ATCC) 3 days after the final immunization. Supernatants (SNs) from the resulting hybridomas were screened for their ability to stain Jurkat cells. Selected hybridomas in terms of stable-staining activity were subjected to subsequent cloning. One clone, #16-15 (rat IgM), was established.

### Cell culture

Human T cells (1 × 10^4^/w) were stimulated with anti-CD3/CD28-coated beads (2 × 10^4^/w, Dynabeads, Invitrogen, Carlsbad, CA). Cells were maintained at 37°C with 5% CO_2_ in RPMI supplemented with 10% fetal bovine serum, penicillin (100 U/ml), and streptomycin (0.1 mg/ml) unless stated otherwise. The proliferation of T cells was assessed by measuring the incorporation of [^3^H]TdR (37 kBq/well) for the final 4 h of a 3-day culture.

### Immunoprecipitation and immunoblotting

Cells were washed twice with PBS, and lysed for 1 h at 4°C in lysis buffer (50 mM Tris–HCl, pH7.5, 150 mM NaCl, 5 mM EDTA, and 1% Triton X-100) supplemented with protease and phosphatase inhibitors (both purchased from Nacalai Tesque, Kyoto, Japan). Cell lysates were then separated from the debris by centrifugation at 20,000*g* for 15 min at 4°C. Protein concentrations were determined by the BCA protein assay (Thermo), 5–20 μg cell lysates were separated by SDS–PAGE under reduced conditions, and proteins were electrotransferred onto PVDF membranes (Millipore, Billerica, MA). After blocking with blocking-one (Nacalai Tesque) in Tris-Buffered saline containing 0.1% Tween 20, the membrane was incubated overnight at 4°C with the indicated primary antibody, washed, and subjected to chemiluminescence detection with the HRP-conjugated secondary antibody with ECL (Millipore). In some experiments, cell lysates (500–1000 μg) were incubated with the indicated primary antibody for 2 h at 4 °C. Immunocomplexes were precipitated with protein A-Sepharose (Sigma, Tokyo, Japan) for 1 h at 4°C. Immunoprecipitates were washed four times with ice-cold wash buffer (50 mM Tris–HCl, pH 7.5, 150 mM NaCl, and 1% Triton X-100). Immunoprecipitated proteins were eluted with sample buffer containing 100 mM DTT and heated for 10 min at 96 °C.

### Plasmid preparation and transfection

The full length of the BEND3 gene was amplified from Jurkat cDNA with KOD-Plus (Toyobo, Osaka, Japan) using the primers 5′-AGACCCAAGCTTATGAACTCAACTGAATTCACCG-3′ and 5′-AATATAGCGGCCGCTCACTTCTCCACTTTCTTTGC-3′. The PCR product was cloned into the HindIII/NotI site of the pCMV-(DYKDDDDK)-N vector (Clontech, Palo Alto, CA), resulting in a FLAG-BEND3 expression plasmid. The C-terminally truncated BEND3 gene was amplified from the plasmid using the following reverse primers: BEND3_1–414_ 5′-AATATAGCGGCCGCTCAGAGGAAGACGGCAAACTCGCC-3′, BEND3_1–420_ 5′-AATATAGCGGCCGCTCAGGGGAAGAGCCGGTGGAGGAG-3′, BEND3_1–425_ 5′-AATATAGCGGCCGCTCAGTGGTCGAAGAGCTCGGGGAAG-3′, BEND3_1–430_ 5′-AATATAGCGGCCGCTCAGTAGCAGCTGTACTGTTCACCC-3′, and subcloned into the pCMV-(DYKDDDDK)-N vector. Transfection of the plasmid prepared above into Jurkat cells was accomplished using the Amaxa Nucleofector system and Cell Line Nucleofector kit (Lonza, Walkersville, MD) according to the manufacturer's protocol.

### Cytokine assays

We analyzed the cytokine content of SNs using a Bio-Plex kit (Bio-Rad, Richmond, CA) following the manufacturer's instructions.

### Immunofluorescence and confocal microscopy

All FACS data were acquired on an FACSCanto II flow cytometer (BD Biosciences) using FACSDiva software. Data were analyzed using Flowjo software (Treestar, San Carlos, CA). Cells were fixed with BD cytofix fixation buffer (BD Biosciences) for 15 min on ice, and were then permeabilized with 0.1% Triton X-100/PBS for 10 min at room temperature for intracellular staining. Cells were stained with #16-15, followed by PE conjugated anti-rat IgM. Images were obtained with a LSM 710 confocal microscope (Carl Zeiss, Oberkochen, Germany).

### Statistical analysis

Data were analyzed using the Student's *t*-test as indicated in the figure legends. The mean is given as ±SD with the following *P*-values being considered significant: *P < 0.05; **P < 0.01.

## SUPPORTING INFORMATION

**Figure S1.** The proportion of memory T cells in (#16-15)^+^ or (#16-15)^−^ T cells. **Figure S2.** Staining of mouse T cells with #16-15.
