# Supplementary material for: Human T cells expressing BEND3 on their surface represent a novel subpopulation that preferentially produces IL-6 and IL-8
Source: Immun Inflamm Dis. 2014 Feb 19;2(1):35–43. doi: 10.1002/iid3.17 (PMC4220666; doi:10.1002/iid3.17)
Supplement: Supplementary file 1 [file iid30002-0035-SD1.ppt]

## Slide 1
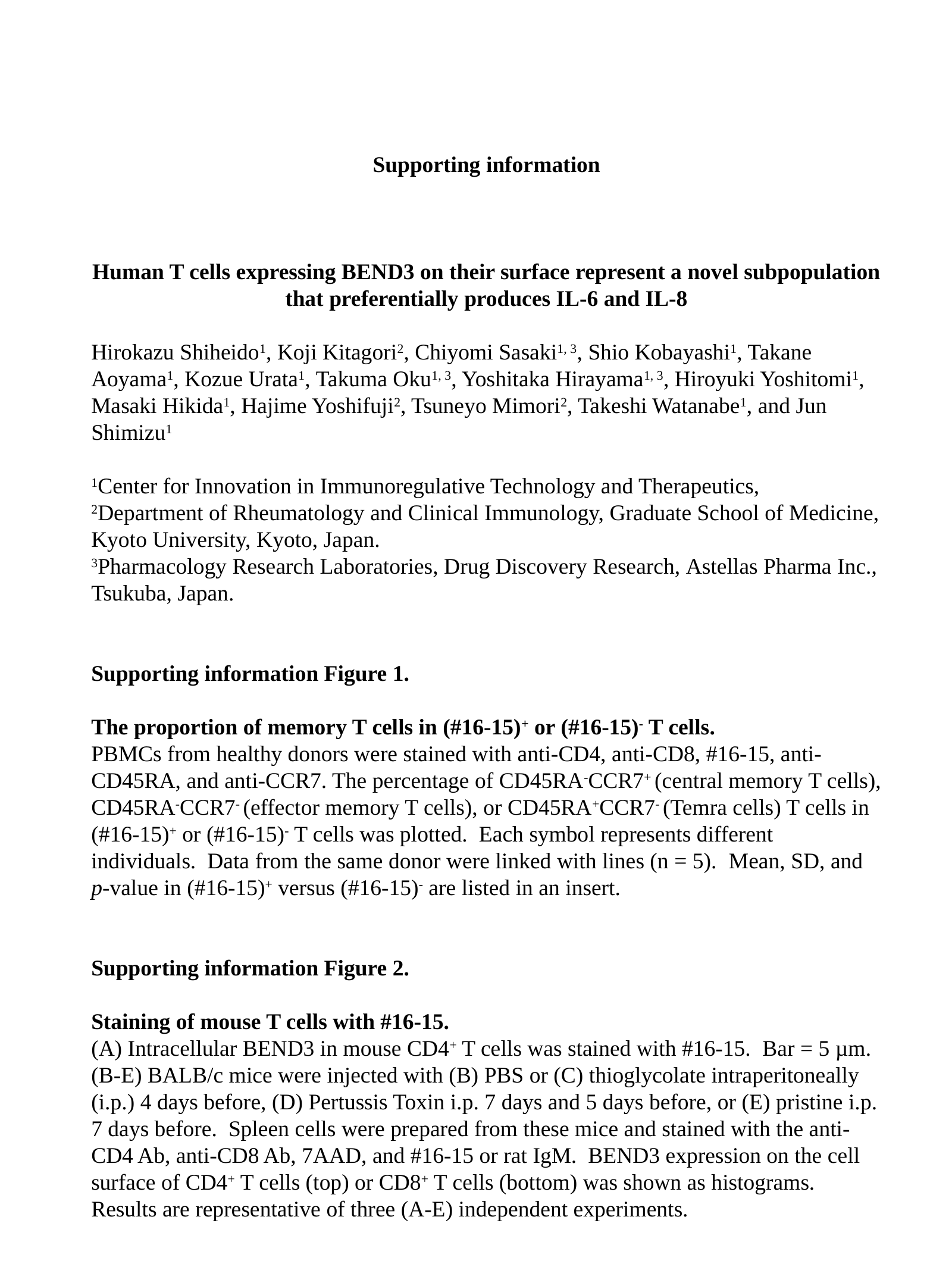

Supporting information
Human T cells expressing BEND3 on their surface represent a novel subpopulation that preferentially produces IL-6 and IL-8
Hirokazu Shiheido1, Koji Kitagori2, Chiyomi Sasaki1, 3, Shio Kobayashi1, Takane Aoyama1, Kozue Urata1, Takuma Oku1, 3, Yoshitaka Hirayama1, 3, Hiroyuki Yoshitomi1, Masaki Hikida1, Hajime Yoshifuji2, Tsuneyo Mimori2, Takeshi Watanabe1, and Jun Shimizu1
1Center for Innovation in Immunoregulative Technology and Therapeutics,
2Department of Rheumatology and Clinical Immunology, Graduate School of Medicine, Kyoto University, Kyoto, Japan.
3Pharmacology Research Laboratories, Drug Discovery Research, Astellas Pharma Inc., Tsukuba, Japan.
Supporting information Figure 1.
The proportion of memory T cells in (#16-15)+ or (#16-15)- T cells.
PBMCs from healthy donors were stained with anti-CD4, anti-CD8, #16-15, anti-CD45RA, and anti-CCR7. The percentage of CD45RA-CCR7+ (central memory T cells), CD45RA-CCR7- (effector memory T cells), or CD45RA+CCR7- (Temra cells) T cells in (#16-15)+ or (#16-15)- T cells was plotted. Each symbol represents different individuals. Data from the same donor were linked with lines (n = 5). Mean, SD, and p-value in (#16-15)+ versus (#16-15)- are listed in an insert.
Supporting information Figure 2.
Staining of mouse T cells with #16-15.
(A) Intracellular BEND3 in mouse CD4+ T cells was stained with #16-15. Bar = 5 µm. (B-E) BALB/c mice were injected with (B) PBS or (C) thioglycolate intraperitoneally (i.p.) 4 days before, (D) Pertussis Toxin i.p. 7 days and 5 days before, or (E) pristine i.p. 7 days before. Spleen cells were prepared from these mice and stained with the anti-CD4 Ab, anti-CD8 Ab, 7AAD, and #16-15 or rat IgM. BEND3 expression on the cell surface of CD4+ T cells (top) or CD8+ T cells (bottom) was shown as histograms. Results are representative of three (A-E) independent experiments.

## Slide 2
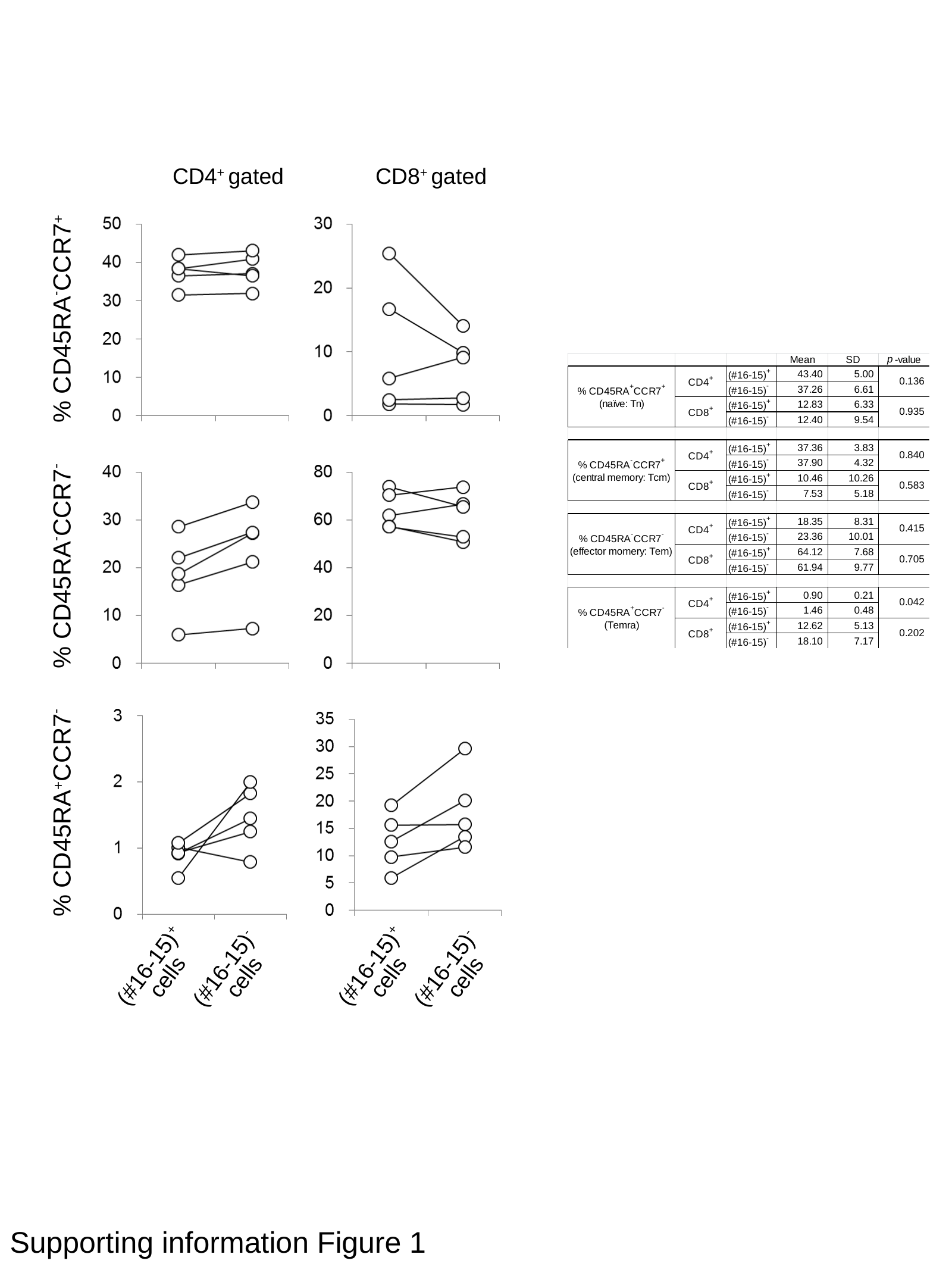

CD4+ gated
CD8+ gated
% CD45RA-CCR7+
% CD45RA-CCR7-
% CD45RA+CCR7-
(#16-15)+
cells
(#16-15)+
cells
(#16-15)-
cells
(#16-15)-
cells
Supporting information Figure 1

## Slide 3
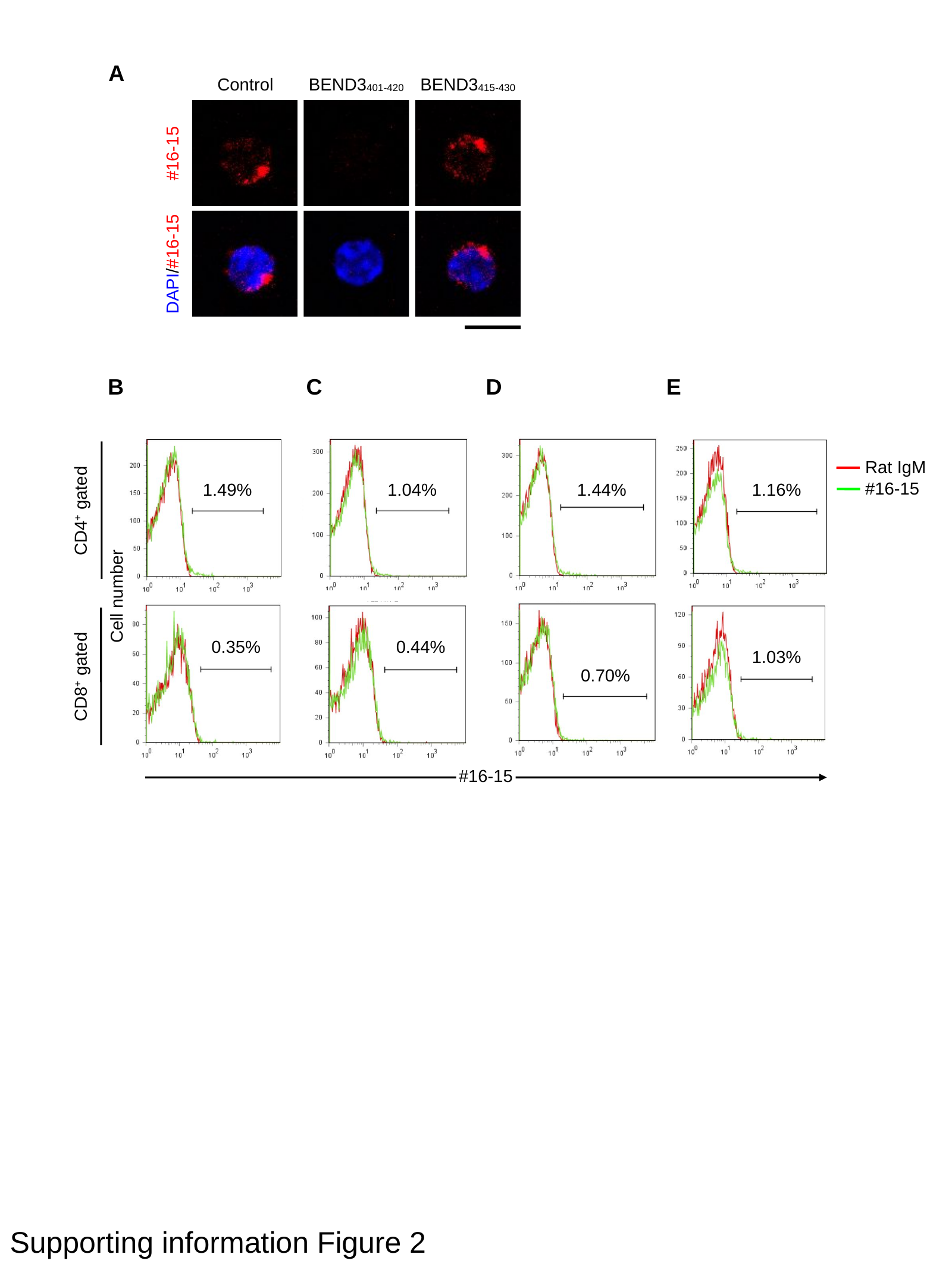

A
Control
BEND3401-420
BEND3415-430
#16-15
DAPI/#16-15
B
C
D
E
Rat IgM
#16-15
1.49%
1.04%
1.44%
1.16%
CD4+ gated
Cell number
0.35%
0.44%
1.03%
CD8+ gated
0.70%
#16-15
Supporting information Figure 2
